# Supplementary material for: Amine recognizing domain in diverse receptors from bacteria and archaea evolved from the universal amino acid sensor
Source: bioRxiv. 2023 Apr 6:2023.04.06.535858. Preprint. [Version 1] doi: 10.1101/2023.04.06.535858 (PMC10104139; doi:10.1101/2023.04.06.535858)
Supplement: Supplement 1 [file media-1.pdf]

Supplementary Material

Amine recognizing domain in diverse receptors from bacteria and archaea evolved from the universal amino acid sensor

Jean Paul Cerna-Vargas, Vadim M. Gumerov, Tino Krell and Igor B. Zhulin

This file contains: Supplementary figures S1 through S6

Supplementary tables S1 through S4

Supplementary references

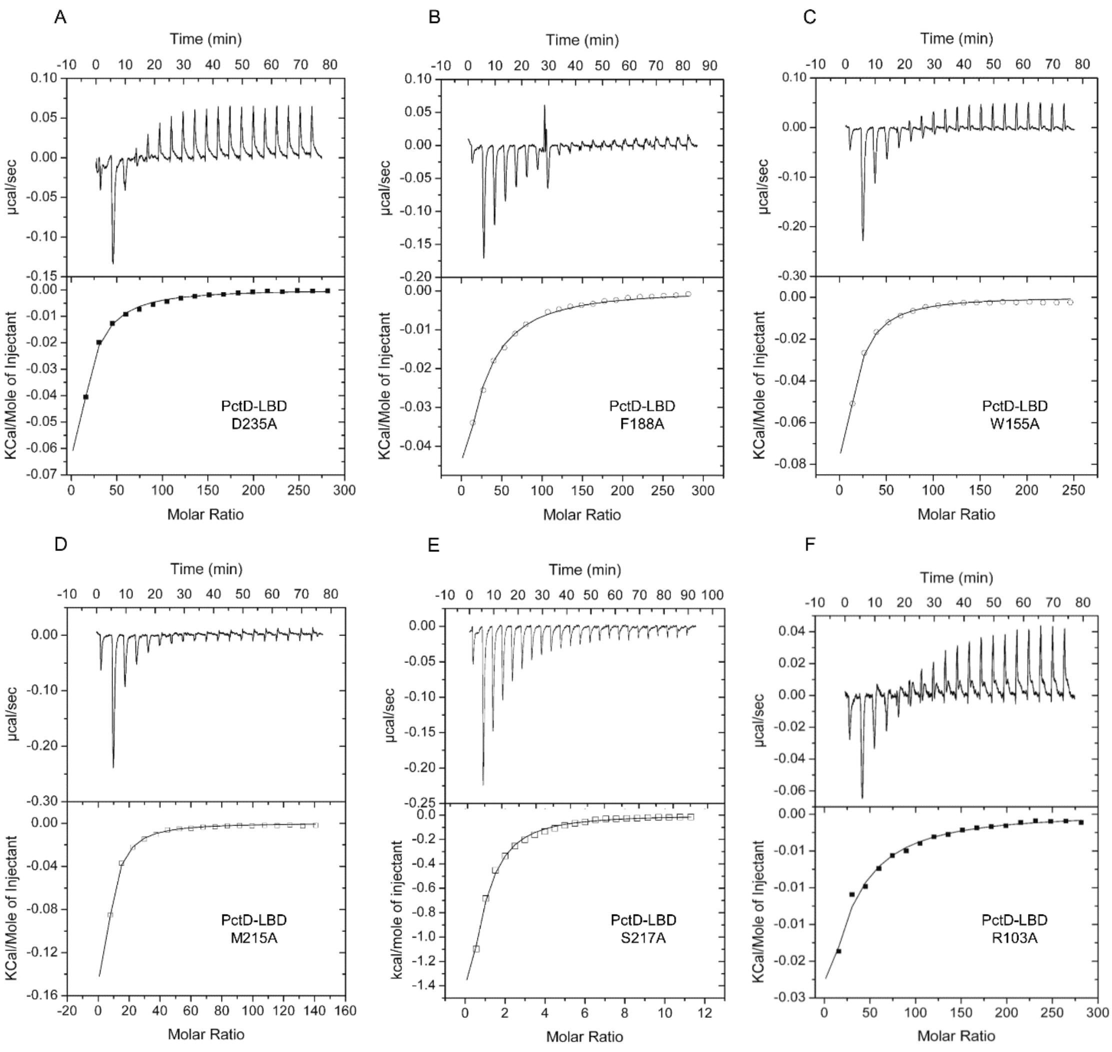

Fig. S1) Microcalorimetric titrations of PctD-LBD mutants with choline. Proteins at 14 to 16 µM were placed into the sample cell and titrated with 9.6 to 14.42 µL aliquots of 1mM (PctD-LBD S217A), 10 mM (PctD-LBD M215A) or 20 mM choline (the remaining mutants) solutions made up in dialysis buffer. Upper panels: raw titration data. Lower panels: integrated, concentration-normalized and dilution heat-corrected peak areas and best fit using the “one–binding site model” of the MicroCal version of ORIGIN. The derived thermodynamic parameters are shown in Table. 1.

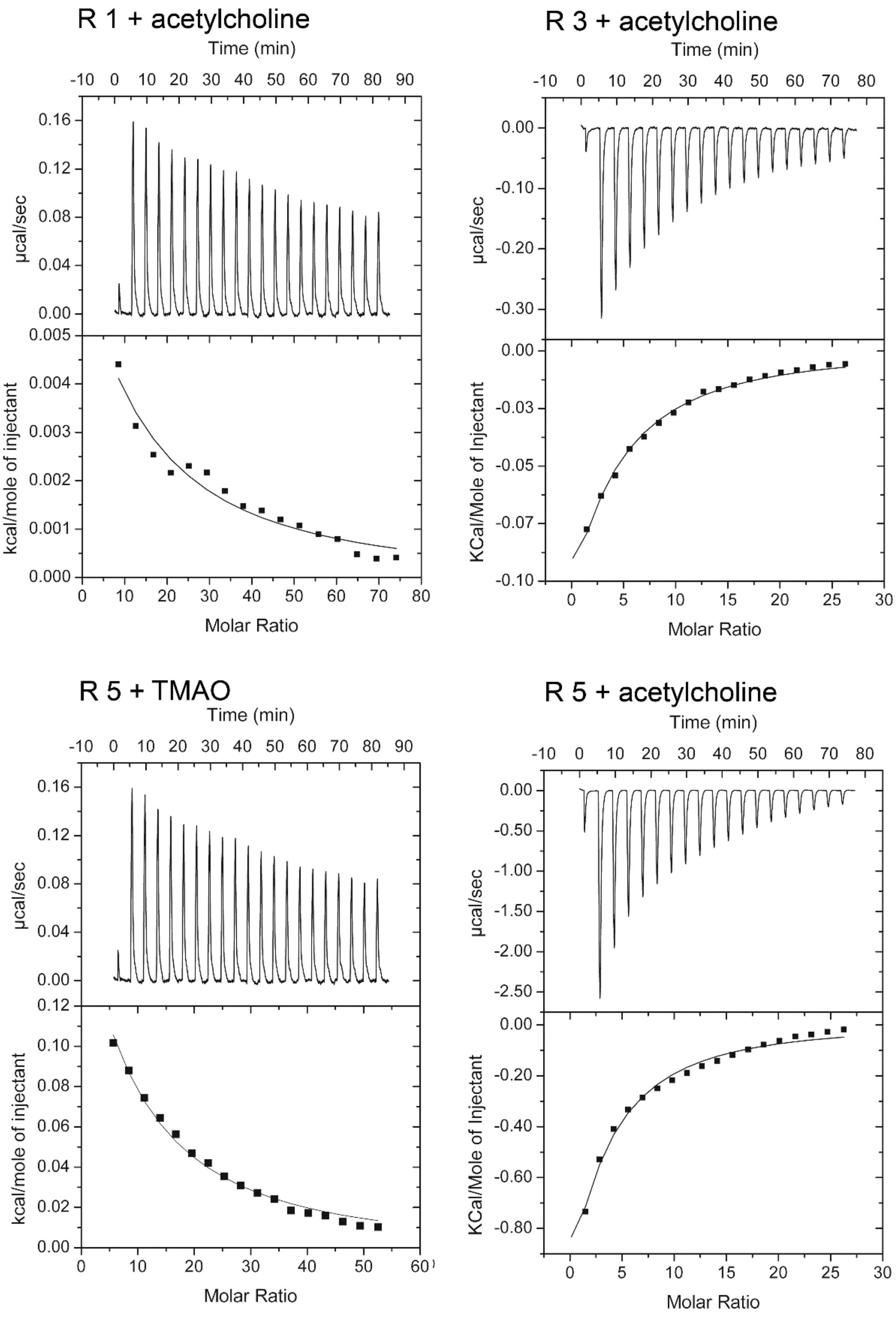

Fig. S2) Microcalorimetric titrations of predicted amine responsive dCache domains with different quaternary amines. Upper panel: Raw data for the titration of 75 µM of protein with 14.42 µl aliquots of 10 to 20 mM of quaternary amines. Lower panel: Concentration-normalized and dilution heat corrected integrated raw data. The line is the best fit using the “One binding site model” of the MicroCal version of ORIGIN.

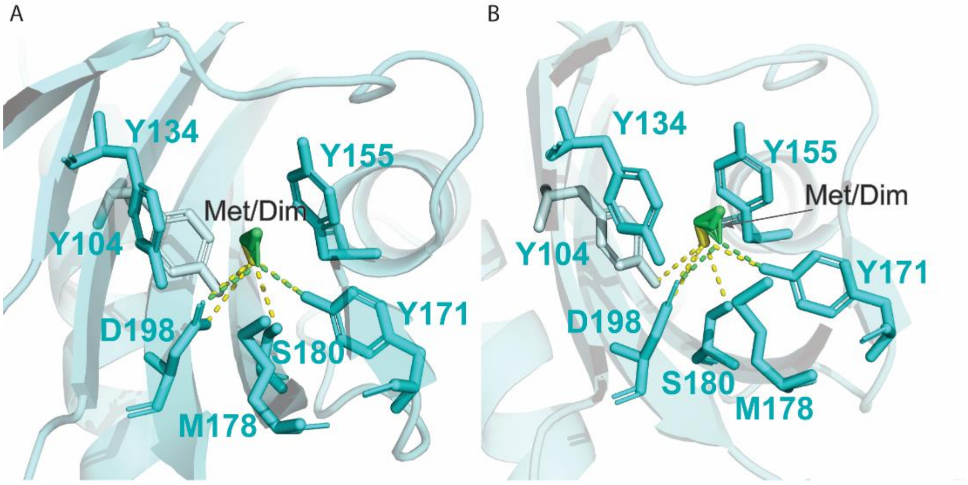

Fig. S3) Ligand binding module of the dCache_1 domain from the archaeon *Methanosarcina mazei* docked with methylamine (Met, in yellow) and dimethylamine (Dim, in green). A and **B** are two slightly different angles. Predicted polar contacts are shown.

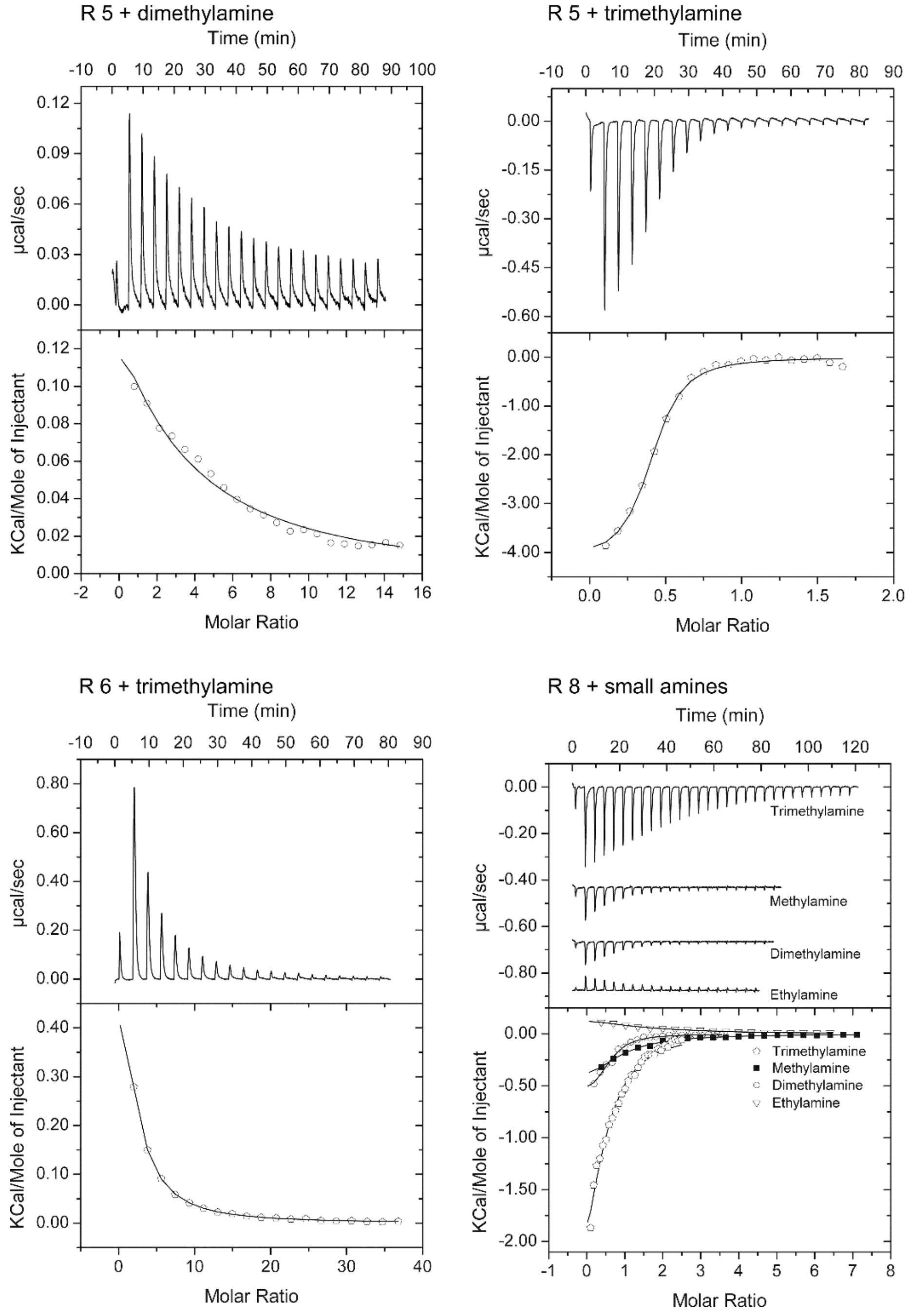

Fig. S4) Microcalorimetric titrations of predicted amine responsive dCache domains with different small amines. Upper panel: Raw data for the titration of 30 to 50 µM of protein with 3.2 to 12.8 µl aliquots of 1 to 10 mM of small amines. Lower panel: Concentration-normalized and dilution heat corrected integrated raw data. The line is the best fit using the “One binding site model” of the MicroCal version of ORIGIN.

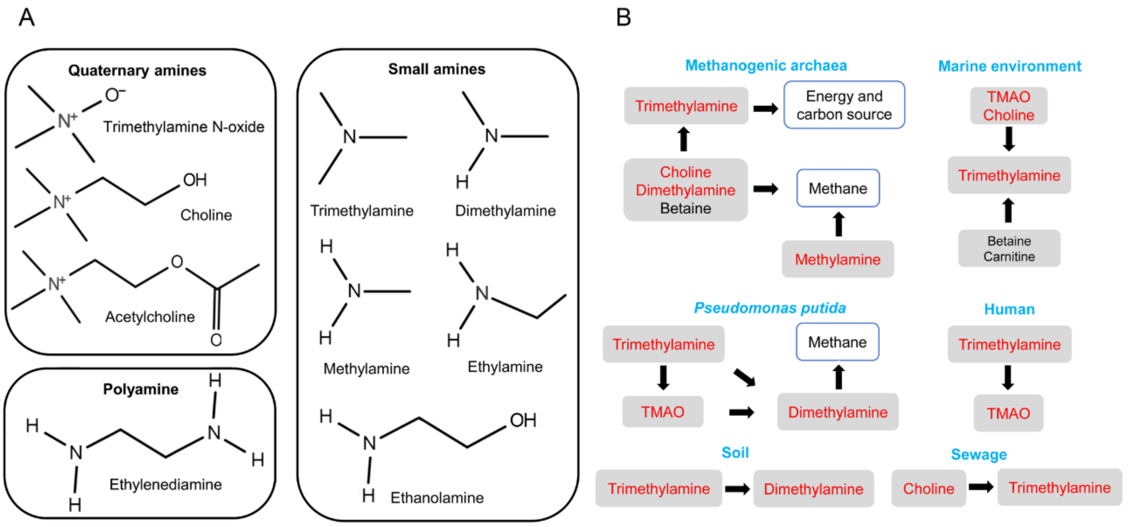

Fig. S5) Structures of ligands recognized by this domain family (A) and major metabolic processes involving these ligands (B). Ligands recognized by members of this domain family are shown in red. Information on the metabolic processes have been obtained from the following sources: methanogenic archaea (1, 2), marine environment (3, 4), *Pseudomonas putida* (5), human (6), soil (7) and sewage(8).

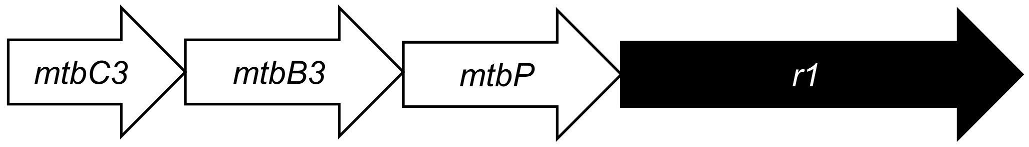

mtbC3: Dimethylamine-corrinoid protein
mtbB3: Dimethylamine-corrinoid methyltransferase
mtbP: APC family permease
r1: Receptor 1

Fig. S6) Genetic environment of the gene encoding R1 (Histidine kinase from *Methanosarcina mazei* S6). The apo-3D structure of the R1-LBD has been reported (9). There is 100 % sequence identity between the above four genes in *M. mazei* S6 and *M. mazei* Gö1. The latter strain was used in a study the assess the effect of trimethylamine on gene transcript levels, showing large increases of *mtbC3* and *mtbB3* transcript levels in the presence of trimethylamine (10).

Table S1) Increases in the midpoint of the thermal unfolding transition of protein (ºc) in the presence of a given ligand as compared to ligand-free protein. Conditions in which binding was observed by isothermal Titration Calorimetry are shown in bold.

Protein	L-Carnitine	Betaine	L-Pro	Methylamine	Ethylamine	Dimethylamine	Trimethylamine	Ethanolamine	Ethylenediamine
R1	0.44	-0.59	0	**3.74**	**2.41**	**12.5**	**8.5**	-0.18	-0.24
R3	-0.35	-0.78	-0.35	0.2	0.1	0.5	0.75	**13.9**	**6.9**
R4	0.20	1.67	1.00	0.59	0.6	0.9	3.84	1	0.8
R5	-0.27	-0.26	-0.53	0.27	0.84	**3.45**	**10.49**	0.85	1.52
R6	1.13	-0.26	0.73	0.2	0.1	5	**4.8**	0.3	-0.2
R7	0	-0.04	-1.83	**6**	0.1	**12**	**9**	**15**	**10**
R8	0.40	-0.6	-1.00	**5.29**	**3.68**	**6.63**	**7.13**	0.2	0.3
R9	0.67	0	0.67	0.2	0.3	-0.1	-0.3	0.1	0.2
R10	0.27	-1.22	0.27	0.19	-0.21	-0.71	-0.48	0.09	0.23
PacA-LBD	nd	nd	nd	0.1	0.2	0.1	**5**	0.1	0.2

Table S2) Strains and plasmids used in this study.

	Relevant characteristics	Reference or source
**Strains**		
Escherichia coli BL21 (DE3)	F^{-} *ompT gal dcm lon hsdS*$_B$($r_B^- m_B^-$) λ(DE3 [*lacI lacUV5-T7p07 ind1 sam7 nin5*]) [*malB*$^+$]$_{K-12}$(λ^S)	(11)
E. coli BL21-AI	F- *ompT hsdS*$_B$ ($r_B^- m_B^-$) *gal dcm araB*::*T7RNAP-tetA*	Invitrogen
**Plasmids**		
pET28_ECA_RS10935 -LBD	Km^R; pET28b(+) derivative containing a DNA fragment encoding PacA-LBD (*Pectobacterium atrosepticum* SCRI1043)	(12)
pET28-PctD-LBD-D235A	Km^R; pET28b(+) derivative containing DNA fragment encoding PctD-LBD D235A	GenScript
pET28- PctD-LBD-F188A	Km^R; pET28b(+) derivative containing DNA fragment encoding PctD-LBD F188A	GenScript
pET28- PctD-LBD-W155A	Km^R; pET28b(+) derivative containing DNA fragment encoding PctD-LBD W155A	GenScript
pET28- PctD-LBD-M215A	Km^R; pET28b(+) derivative containing DNA fragment encoding PctD-LBD M215A	GenScript
pET28- PctD-LBD-S217A	Km^R; pET28b(+) derivative containing DNA fragment encoding PctD-LBD S217A	GenScript
pET28- PctD-LBD-R103A	Km^R; pET28b(+) derivative containing DNA fragment encoding PctD-LBD R103A	GenScript
pET28-R1-LBD	Km^R; pET28b(+) derivative containing DNA fragment encoding R1-LBD	GenScript
pET28-R2-LBD	Km^R; pET28b(+) derivative containing DNA fragment encoding R2-LBD	GenScript
pET28-R3-LBD	Km^R; pET28b(+) derivative containing DNA fragment encoding R3-LBD	GenScript
pET28-R4-LBD	Km^R; pET28b(+) derivative containing DNA fragment encoding R4-LBD	GenScript
pET28-R5-LBD	Km^R; pET28b(+) derivative containing DNA fragment encoding R5-LBD	GenScript
pET28-R6-LBD	Km^R; pET28b(+) derivative containing DNA fragment encoding R6-LBD	GenScript
pET28-R7-LBD	Km^R; pET28b(+) derivative containing DNA fragment encoding R7-LBD	GenScript
pET28-R8-LBD	Km^R; pET28b(+) derivative containing DNA fragment encoding R8-LBD	GenScript
pET28-R9-LBD	Km^R; pET28b(+) derivative containing DNA fragment encoding R9-LBD	GenScript
pET28-R10-LBD	Km^R; pET28b(+) derivative containing DNA fragment encoding R10-LBD	GenScript

Km = Kanamycin

Table S3) Sequences of recombinant proteins used in this study.

Protein	Sequence
**PacA-LBD**	MGSSHHHHHHSSGLVPRGSHMSWQSSSEQKSLAERYLQQIAQSEALRIQQE<br>LNYARDVAHNLGQGLAALPSAGIKDRAVVDKMMEYALRDNPEYLSISVIFE<br>ENVFDGRDAEFADQPGQAPKGRYAWFVDRDQAGNYAMHPLLSYLTPGQGDY<br>YLLPQKSQKDTLIEPYTYAYNGVPTLLTSVAAPIVSQGKLWGVVTSDISLA<br>SLQQKINQIKPWEGGGYAMLLSSAGKVISYPDKSQTSKAWQGPTDNFTSSV<br>VQHDDAILGEQALVTWQPVTIGNSTEKWYLGIVVPVSQVMAASERQL
**PctD-LBD R103A**	MGSSHHHHHHSSGLVPRGSHMAGARTQELVQQRTQGLLEKVINERLVALAR<br>AQVSQIQRELEYPLTVVHGLANSTRLLGEPGADGMPQLNASADEISALLRS<br>TVQNNPKLLDTFMAWEPNAFDTDAAFAGQPGKGYGPDGRYLPWWYRGADGK<br>PIVEAMADSIDSEKLLPTGVRENEFYACPKENKRPCIIDPAPYEMGGKTVM<br>MSSFNVPIMVGDQFRGAVGADLSLAFIQDLLKRADQQLYDGAGEMALIASN<br>GRLVAYTRDDSKLGEPAGSVLDGNEVDNLKNLTVDQPLYDIDAEHGHIELF<br>LPFTIADSGVRWTLMLQIPQAAVFGELQQLQGELSDQRQQDILGM
**PctD-LBD W155A**	MGSSHHHHHHSSGLVPRGSHMAGARTQELVQQRTQGLLEKVINERLVALAR<br>AQVSQIQRELEYPLTVVHGLANSTRLLGEPGADGMPQLNASRDEISALLRS<br>TVQNNPKLLDTFMAWEPNAFDTDAAFAGQPGKGYGPDGRYLPAWYRGADGK<br>PIVEAMADSIDSEKLLPTGVRENEFYACPKENKRPCIIDPAPYEMGGKTVM<br>MSSFNVPIMVGDQFRGAVGADLSLAFIQDLLKRADQQLYDGAGEMALIASN<br>GRLVAYTRDDSKLGEPAGSVLDGNEVDNLKNLTVDQPLYDIDAEHGHIELF<br>LPFTIADSGVRWTLMLQIPQAAVFGELQQLQGELSDQRQQDILGM
**PctD-LBD F188A**	MGSSHHHHHHSSGLVPRGSHMAGARTQELVQQRTQGLLEKVINERLVALAR<br>AQVSQIQRELEYPLTVVHGLANSTRLLGEPGADGMPQLNASRDEISALLRS<br>TVQNNPKLLDTFMAWEPNAFDTDAAFAGQPGKGYGPDGRYLPWWYRGADGK<br>PIVEAMADSIDSEKLLPTGVRENEAYACPKENKRPCIIDPAPYEMGGKTVM<br>MSSFNVPIMVGDQFRGAVGADLSLAFIQDLLKRADQQLYDGAGEMALIASN<br>GRLVAYTRDDSKLGEPAGSVLDGNEVDNLKNLTVDQPLYDIDAEHGHIELF<br>LPFTIADSGVRWTLMLQIPQAAVFGELQQLQGELSDQRQQDILGM
**PctD-LBD M215A**	MGSSHHHHHHSSGLVPRGSHMAGARTQELVQQRTQGLLEKVINERLVALAR<br>AQVSQIQRELEYPLTVVHGLANSTRLLGEPGADGMPQLNASRDEISALLRS<br>TVQNNPKLLDTFMAWEPNAFDTDAAFAGQPGKGYGPDGRYLPWWYRGADGK<br>PIVEAMADSIDSEKLLPTGVRENEFYACPKENKRPCIIDPAPYEMGGKTVM<br>ASSFNVPIMVGDQFRGAVGADLSLAFIQDLLKRADQQLYDGAGEMALIASN<br>GRLVAYTRDDSKLGEPAGSVLDGNEVDNLKNLTVDQPLYDIDAEHGHIELF<br>LPFTIADSGVRWTLMLQIPQAAVFGELQQLQGELSDQRQQDILGM
**PctD-LBD S217A**	MGSSHHHHHHSSGLVPRGSHMAGARTQELVQQRTQGLLEKVINERLVALAR<br>AQVSQIQRELEYPLTVVHGLANSTRLLGEPGADGMPQLNASRDEISALLRS<br>TVQNNPKLLDTFMAWEPNAFDTDAAFAGQPGKGYGPDGRYLPWWYRGADGK<br>PIVEAMADSIDSEKLLPTGVRENEFYACPKENKRPCIIDPAPYEMGGKTVM<br>MSAFNVPIMVGDQFRGAVGADLSLAFIQDLLKRADQQLYDGAGEMALIASN<br>GRLVAYTRDDSKLGEPAGSVLDGNEVDNLKNLTVDQPLYDIDAEHGHIELF<br>LPFTIADSGVRWTLMLQIPQAAVFGELQQLQGELSDQRQQDILGM
**PctD-LBD D235A**	MGSSHHHHHHSSGLVPRGSHMAGARTQELVQQRTQGLLEKVINERLVALAR<br>AQVSQIQRELEYPLTVVHGLANSTRLLGEPGADGMPQLNASRDEISALLRS<br>TVQNNPKLLDTFMAWEPNAFDTDAAFAGQPGKGYGPDGRYLPWWYRGADGK<br>PIVEAMADSIDSEKLLPTGVRENEFYACPKENKRPCIIDPAPYEMGGKTVM<br>MSSFNVPIMVGDQFRGAVGAALSLAFIQDLLKRADQQLYDGAGEMALIASN<br>GRLVAYTRDDSKLGEPAGSVLDGNEVDNLKNLTVDQPLYDIDAEHGHIELF<br>LPFTIADSGVRWTLMLQIPQAAVFGELQQLQGELSDQRQQDILGM
**R1**	MGSSHHHHHHSSGLVPRGSHMTTQEEKLAYQQSVEMASNYANQFDADMKAN<br>LAIARTISTTMESYETADRDEALLILENLLRDNPHLLGTYVAFEPDAFDGK<br>DAEYTNSPAHDGTGRFVPYWNKMNGTASVAPLLHYDSSDYYQLPKATEKDV<br>LTEPYFYEGVFMVSYVSPIMKEGEFAGIGGVDVSLEYVDEVVSKVRTFDTG

	YAFMVSNSGVILSHPTHKDWIGKKDLYDFGGEELEKASRDIKNGIGGHLET<br>ADPTTGKTVILFYEPVETGDFAFVLVVPKEEMLAGVADLRER
**R2**	MGSSHHHHHHSSGLVPRGSHMQLMKLYDVSLRQGELVAQNQSNAYTTKMSI<br>ETNDALIRLEGLQQSLQQMKEYNMTDRSEAVRLIENFVREQPYILGVFTVW<br>EPNAFDNQDGNFRNKSSYDDDTGRFVPYIVRQGDKIVAYPNKNYENIGDGD<br>YYQIPKRTKKFALMEPYYYDINGERILISSFVYPILDEQGKFLGVVGADIS<br>LDMVQQEVEKIRPMGGYATMITAGDSYLANGFDRALVSKPYLPLPKGESLE<br>ELKEQALTIMYTSDPMLGGTVMRLLNPIHIKDQTWYFETIIPKGNMLKDYY<br>KGLSNT
**R3**	MGSSHHHHHHSSGLVPRGSHMHTSSKTTLMQEARADAANLTLASIRKIEGT<br>LASVEAIPGLLAFSYGKNKPTASAISTDLLGFILFNSAVYGSCVAYEPYAF<br>DRDVEFFAPYAYMPGGRPMFTYLSADYNYPQADWFLIPKEIRRPIWSEPYF<br>DEGGGNVVMSTYSIPFFREEDGRKRFLGVVTADISLEWLRTFIKSISIYQS<br>GYAFLLSRNGVFLSHPNDQFIMRESIFSLAETHSSKVLRDIGKKMVQGETG<br>FVRLPEFVMGEPAWLSYAPVSNSDWSMGLVIPEAEMFQGLEGLSRE
**R4**	MGSSHHHHHHSSGLVPRGSHMSNRSIEMAQKDAFSLAQETADKYKNAIIAE<br>LQGARITAETFSTVFETLKDYNLTDRDMMNDILKNALANKEYITAFCIAYD<br>PNAMDGKDAQYAGQGPAYDETGRYAPYWNKLGGNIDVEFLPDIDSEDWYIV<br>PKAERHEYITDPYPYGLQGRTVMLASLIFPIIHSDKFIGIISSDIVLDKLQ<br>EMVDKVNPHGQEGYTEIISHSGAVIAHPNKDYLGKDLEETLVEGQSRLQHI<br>DEIKSAINSGEMYISTGKNFYTVYMPIQFSSVTNPWSVAVSIPMAKILANA<br>DSIRNY
**R5**	MGSSHHHHHHSSGLVPRGSHMTYNSLKTATVSSTEISNQMAVTYANQVVDK<br>MDDAMSAARSLAHALSGVIGKNVSRQAIQQMAGSILLGDEDFLGYTVCFEP<br>NAYDAKDAFFANKPGHDNTGRFVSYMTKNGSGGFVVEPLVDYENESAAPWY<br>WIPMRTMKEFVTEPLMYPIQGKNVYMVSFMCPIITNGKFVGVTGVDLSINY<br>LQDMVVKANVFDGHGNFDIVSHQGVFAANSGNPDFVGKNILEQKNIGAEDQ<br>LVDIEKGNSLTRIDNGILKAFVPVIVGRCPTAWQVSISVPVDYITQEARAQ<br>MIYQ
**R6**	MGSSHHHHHHSSGLVPRGSHMRVVHSARQEANALSRTKAQAIGAEMAHRLG<br>RAIGTARTLSEALEGILAEGHPSRAQADAMLRGSLEGNTDYIGVWTLWEPN<br>AFDGRDADYVNKPGHDATGRYIAYWNRGSGKVIVEPLVDYTTEGAGDYYLL<br>AKHSNQETVLEPYIYKVAGRDVLMTSLVVPVNRADGTFAGVVGVDLPLETL<br>GAEIAKVKVGETGYAALVSNTGIYAAHPRAERLGKPMKDTDPWVVPFLGNL<br>KKGEAFETESFSRTLNDMTYRFGVPVRIGSSSTPWCVSITIRESEVLAGAW<br>KLRNT
**R7**	MGSSHHHHHHSSGLVPRGSHMSYINARNEALNAAQKRAQIVAKNYSKEISD<br>ELGQAITVAENLGSMLKGRIRNENATLTRDEVSEIFKNALADNPQFIGISI<br>AFEPNAFDSLDAQFDGDSRYYEKGQFATYFVRGNLNSGKDMYSTITQEPLR<br>DLEISDYYIVPKQTLSNVMIEPYIYEVQGKEVLMTTCSSPIVIDGKYYGVV<br>GVDIEVDFIKELVKGSENDMEFKDILIISSKGNIVGSKYEMAYNQDENLKE<br>EILHFKQGSHVEYSNGIFDVYELINIRDIDDKWGIKLSVDQKTIMGSASRL<br>LANQ
**R8**	MGSSHHHHHHSSGLVPRGSHMTTKSGSDIETLAFQSGEQLGHRYGEMVHAR<br>LGNAMEAGRFIATSLVGLKAAGRTDREQLSIWLKSIAEANPDFLGVWVGME<br>PNALDGRDAEFANKPGSDASGRFLPYWNRGSGTVALESLVGYDEPGSDGAY<br>YQIPKRTGHAMVVEPYSYTVAGRKVLMVSMSVPIVENGRVIGVAGIDLSTD<br>GIWSMLKTVKPFDSGSIHLISNDGVWAGHPDSERMGQPIGKSDPALDAAKP<br>AIRAGRSFEQMSVADGQPVKQLFLPVTVAGTETPWSLLVNLPLDKINAPVR<br>ELRNAT
**R9**	MGSSHHHHHHSSGLVPRGSHMYNARASQQTAKLQSSESVIDKSQQLLQTGA<br>LLNATEISEYLSEAIYRAEMLAANALFLKNNSEENFGESEALRTSLDEMVR<br>KSVLGFDTIEGAYLVFRPNMLDSEDSNYVNADYVGSNDIGQFAAYWTKAAN<br>GQNVISRVLTQAQLTEESNKERFVCPIEQASPCITSPRMVEFETERYLATS<br>LSVPILIDGVAIGFYGIDLTLAPLIGITQKSDNNLFDGQGKVSIVSENNAL

	VASDADFLTLGETFQSENLSRSTVSSLLQAGQVNTQWSEDGQWLVVFAPTK VANQNWGVIFEMPRQSVMQDAEQLDILLTEQLERGIRSE
**R10**	MGSSHHHHHHSSGLVPRGSHMTTLVYNNDKKSALLYMESLAAEKANIAKLE METALETARTLASVFSTWENIPVEERRTLFSGILKTVVEKNEDFQGAWTCW EENTLDASDSFYKGLPGYDETGRFIPYWYRSDSGRIEYEPLTGYTQPGEGN YYLVPLNNKKEAAAEPYIYELKGKPRWLTSLSVPIYDNANRVAGIVGINLS LDHLQSHLSDLVFFDTGFGRLVSAEGLVVTHPDRDRIGKIIGEFVKDTGQA LINSIKGGEATSGEAWSESLESMTTKTNVPFSIGRTETNWFYGTVVPSHEL YANALGFAK

Table S4) Buffers used in the purification and analysis of proteins.

Prot.	Purification buffer A	Purification buffer B	Analysis buffer
R1	30 mM Tris, 300 mM NaCl, 5 % glycerol (vol/vol), 10 mM imidazole, pH 7.5	30 mM Tris, 300 mM NaCl, 5 % glycerol (vol/vol), 500 mM imidazole, pH 7.5	5 mM Tris, 5 mM MES, 5 mM PIPES, 150 mM NaCl, 10% (vol/vol) glycerol, pH 7.5
R2	30 mM Tris, 300 mM NaCl, 5 % glycerol (vol/vol), 10 mM imidazole, pH 7.5	30 mM Tris, 300 mM NaCl, 5 % glycerol (vol/vol), 500 mM imidazole, pH 7.5	5 mM Tris, 5 mM MES, 5 mM PIPES, 150 mM NaCl, 10% (vol/vol) glycerol, pH 7.5
R3	30 mM Tris, 300 mM NaCl, 5 % glycerol (vol/vol), 10 mM imidazole, 0.1 mM EDTA, 5 mM β-mercaptoethanol, pH 7.5	30 mM Tris, 300 mM NaCl, 5 % glycerol (vol/vol), 500 mM imidazole, 0.1 mM EDTA, 5 mM β-mercaptoethanol, pH 7.5	5 mM Tris, 5 mM MES, 5 mM PIPES, 150 mM NaCl, 10% (vol/vol) glycerol, 0.1 mM EDTA, 5 mM β-mercaptoethanol, pH 8.0
R4	30 mM Tris, 300 mM NaCl, 5 % glycerol (vol/vol), 10 mM imidazole, 0.1 mM EDTA, 5 mM β-mercaptoethanol, pH 7.5	30 mM Tris, 300 mM NaCl, 5 % glycerol (vol/vol), 500 mM imidazole, 0.1 mM EDTA, 5 mM β-mercaptoethanol, pH 7.5	5 mM Tris, 5 mM MES, 5 mM PIPES, 150 mM NaCl, 10% (vol/vol) glycerol, 0.1 mM EDTA, 5 mM β-mercaptoethanol, pH 8.0
R5	30 mM Tris, 300 mM NaCl, 5 % glycerol (vol/vol), 10 mM imidazole, 0.1 mM EDTA, 5 mM β-mercaptoethanol, pH 7.5	30 mM Tris, 300 mM NaCl, 5 % glycerol (vol/vol), 500 mM imidazole, 0.1 mM EDTA, 5 mM β-mercaptoethanol, pH 7.5	5 mM Tris, 5 mM MES, 5 mM PIPES, 150 mM NaCl, 10% (vol/vol) glycerol, 0.1 mM EDTA, 5 mM β-mercaptoethanol, pH 8.0
R6	30 mM Tris, 300 mM NaCl, 5 % glycerol (vol/vol), 10 mM imidazole, 0.1 mM EDTA, 5 mM β-mercaptoethanol, pH 7.5	30 mM Tris, 300 mM NaCl, 5 % glycerol (vol/vol), 500 mM imidazole, 0.1 mM EDTA, 5 mM β-mercaptoethanol, pH 7.5	5 mM Tris, 5 mM MES, 5 mM PIPES, 150 mM NaCl, 10% (vol/vol) glycerol, 0.1 mM EDTA, 5 mM β-mercaptoethanol, pH 8.0
R7	30 mM Tris, 300 mM NaCl, 5 % glycerol (vol/vol), 10 mM imidazole, 0.1 mM EDTA, 5 mM β-mercaptoethanol, pH 7.5	30 mM Tris, 300 mM NaCl, 5 % glycerol (vol/vol), 500 mM imidazole, 0.1 mM EDTA, 5 mM β-mercaptoethanol, pH 7.5	5 mM Tris, 5 mM MES, 5 mM PIPES, 150 mM NaCl, 10% (vol/vol) glycerol, 0.1 mM EDTA, 5 mM β-mercaptoethanol, pH 8.0
R8	30 mM Tris, 300 mM NaCl, 5 % glycerol (vol/vol), 10 mM imidazole, pH 7.5	30 mM Tris, 300 mM NaCl, 5 % glycerol (vol/vol), 500 mM imidazole, pH 7.5	5 mM Tris, 5 mM MES, 5 mM PIPES, 150 mM NaCl, 10% (vol/vol) glycerol, pH 7.5
R9	30 mM Tris, 300 mM NaCl, 5 % glycerol (vol/vol), 10 mM imidazole, pH 7.5	30 mM Tris, 300 mM NaCl, 5 % glycerol (vol/vol), 500 mM imidazole, pH 7.5	5 mM Tris, 5 mM MES, 5 mM PIPES, 150 mM NaCl, 10% (vol/vol) glycerol, pH 7.5

R10	30 mM Tris, 300 mM NaCl, 5 % glycerol (vol/vol), 10 mM imidazole, 0.1 mM EDTA, 5 mM β-mercaptoethanol, pH 7.5	30 mM Tris, 300 mM NaCl, 5 % glycerol (vol/vol), 500 mM imidazole, 0.1 mM EDTA, 5 mM β-mercaptoethanol, pH 7.5	5 mM Tris, 5 mM MES, 5 mM PIPES, 150 mM NaCl, 10% (vol/vol) glycerol, pH 7.5

References

1. Schorn S, Ahmerkamp S, Bullock E, Weber M, Lott C, Liebeke M, Lavik G, Kuypers MMM, Graf JS, Milucka J. 2022. Diverse methylotrophic methanogenic archaea cause high methane emissions from seagrass meadows. Proc Natl Acad Sci U S A 119:e2106628119.
2. Welte C, Deppenmeier U. 2014. Bioenergetics and anaerobic respiratory chains of aceticlastic methanogens. Biochim Biophys Acta 1837:1130–1147.
3. Lidbury IDEA, Murrell JC, Chen Y. 2015. Trimethylamine and trimethylamine N-oxide are supplementary energy sources for a marine heterotrophic bacterium: implications for marine carbon and nitrogen cycling. ISME J 9:760–769.
4. Zhu Y, Jameson E, Crosatti M, Schäfer H, Rajakumar K, Bugg TDH, Chen Y. 2014. Carnitine metabolism to trimethylamine by an unusual Rieske-type oxygenase from human microbiota. Proc Natl Acad Sci U S A 111:4268–4273.
5. Liffourrena AS, Salvano MA, Lucchesi GI. 2010. Pseudomonas putida A ATCC 12633 oxidizes trimethylamine aerobically via two different pathways. Arch Microbiol 192:471–476.
6. Craciun S, Balskus EP. 2012. Microbial conversion of choline to trimethylamine requires a glycyl radical enzyme. Proc Natl Acad Sci U S A 109:21307–21312.
7. Tate RL, Alexander M. 1976. Microbial formation and degradation of dimethylamine. Appl Environ Microbiol 31:399–403.
8. Thomas JM, Alexander M. 1981. Microbial formation of secondary and tertiary amines in municipal sewage. Appl Environ Microbiol 42:461–463.
9. Zhang Z, Hendrickson WA. 2010. Structural characterization of the predominant family of histidine kinase sensor domains. Journal of molecular biology 400:335–53.
10. Krätzer C, Carini P, Hovey R, Deppenmeier U. 2009. Transcriptional profiling of methyltransferase genes during growth of Methanosarcina mazei on trimethylamine. J Bacteriol 191:5108–5115.
11. Jeong H, Barbe V, Lee CH, Vallenet D, Yu DS, Choi SH, Couloux A, Lee SW, Yoon SH, Cattolico L, Hur CG, Park HS, Segurens B, Kim SC, Oh TK, Lenski RE, Studier FW, Daegelen P, Kim JF. 2009. Genome sequences of Escherichia coli B strains REL606 and BL21(DE3). Journal of molecular biology 394:644–52.
12. Matilla MA, Velando F, Tajuelo A, Martín-Mora D, Xu W, Sourjik V, Gavira JA, Krell T. 2022. Chemotaxis of the Human Pathogen Pseudomonas aeruginosa to the Neurotransmitter Acetylcholine. mBio 13:e0345821.
